# The Thirst Symptom

**Published:** 1885-07

**Authors:** 


### THE THIRST SYMPTOM.

By S. L.

Drs. Hughes and Berridge quarrel about the thirst symptom of *Arsenicum*, and Dr. Proctor sets this matter right in the May number of the *Homœopathic World*, where he agrees with Dr. Hughes that "desire for drink, but inability from the irritable state of the stomach to take more than a small quantity at a time," is a frequent symptom of gastritis, and so might truly call for Arsenicum, while in inflammations occurring elsewhere and in general fevers the thirst may be as insatiable as possible without forbidding its employment. As Arsenicum, besides being a local irritant, is pre-eminently a fever-producer, we should *a priori* expect to find in its fever symptoms a desire for large quantities of water. In cholera, again, where Arsenicum is often useful, the demand for water is excessive; this time, however, not from the pyrexia, but the draining off of the fluids of the body. In each case there is a great systemic demand, and Arsenicum may be found to fit exactly.

During this whole dispute we felt astonished that such a close student of materia medica as Dr. Berridge is known to be could doubt the alternate action of drugs, when, in fact, the very alternation is the bugbear which frightens our students till they get into the habit of reading between the lines, studying the totality of symptoms—in other words, the picture of the diseased state—and then selecting the suitable remedy.

To clear up the case, we studied several drugs in relation to the thirst symptom, and find this alternation in nearly every drug, thus:

*Belladonna.*—Allen, II, 97, quotes: Excessive thirst for cold water; desire to drink from large vessels and a great deal at a time. And again: Great thirst, but unable to satisfy it on account of inability to swallow; tormented with burning thirst; craves drink from time to time, but repels it when offered; aversion to all fluids, so that she behaves frightfully at the sight of them. Let us compare these antagonistic symptoms with those in the *Guiding Symptoms*, and we learn that in the acute inflammatory states, with their unequal circulation, the thirst is assuaged by large drinks, except where the throat is inflammatorily or spasmodically affected, and where the patient would drink if it were not too painful. We even find under Belladonna the symptom given with two strokes: fever heat, now and then sweat; they often ask for drink, but do not take much.

*Apis mellifica* is usually put down as the great remedy for thirstlessness, and still Allen, I, 408, gives us: Burning thirst —seems to rise from the stomach—with dryness in the throat; great thirst (when working at night) after diarrhœa. And in the *Guiding Symptoms*, I, 397, we read of insatiable thirst—thirst so violent that she would like to drink all the time (typhoid); no appetite, but much thirst; drinks often and a little at a time; no thirst even during heat or sweat, but thirst always during the chill.

Of *Pulsatilla* Carroll Dunham teaches (Lectures, II, 63): An almost complete absence of thirst is characteristic of Pulsatilla, yet in fevers where heat follows the chilliness, if it be only a sensation of heat with no objective warmth, there is no thirst; but if the heat be, as it sometimes is, both objective and subjective, it is then attended by thirst. Reichert also calls attention to the fact that though the Pulsatilla symptoms generally are not attended by thirst, yet sometimes thirst is present when the hot stage is strongly marked, and he has had excellent success in puerperal fever and other fevers when thirst was present, the mass of the symptoms having indicated Pulsatilla.

But the clinique corresponds fully with the provings; Allen, VIII, 216: Very violent thirst, especially for beer, after the disappearance of the fever; thirst, without heat and without sweat, immediately after lying down in bed; he longs to drink something invigorating and strengthening.

Similar symptoms we find in the related and still so different *Cyclamen*. No thirst the whole day, but it occurs in the evening as the hands and face become warm; absence of thirst for four days, after which it returned, and was at times greater than in health; thirst so great that she drank eight glasses of water (Allen, IV, 54). The fever, says Carroll Dunham (II, 91), of Cyclamen is partial in all its stages; chill predominates; the heat occurs at evening and is without thirst.

Under our great polychrest, *Sepia*, we find the same different states. Allen, VIII, 619, gives us here: Complete thirstlessness, lasting eleven days; no desire for water at all, even at meal times. And again: Sudden and uncontrollable thirst; must have very cold water, especially in the evening; very thirsty for cold water, drinking much and often; a great desire for water, drinking a glassful at a time—a very unusual thing for me. We easily understand this great thirst, for Dunham remarks that in the fever of Sepia chilliness predominates, but, like the heat, is fugitive and transient; perspiration is copious, especially at night, conjoined with great weakness. If, as Deitmeyer teaches, Sepia retards the circulation and causes an overloading of the vascular system with venous blood, or with blood more or less resembling venous, we can easily understand the thirst of Sepia for the removal of this venous stagnation.

Let this suffice. We could carry the subject over many pages, and still not exhaust it. Mere symptom-covering will not do; we must also know whether the action of the drug at our examination can be related to its primary or secondary effect. Let us not boast of our knowledge, for we have hardly yet digested the very rudiments of therapeutics.

*Pax vobiscum;* let that be our mode of dealing with our apparent opponents, and it is most honorable to acknowledge an error, for every one of us is liable to err.

High or low? is Hale's law of dosage true? *Chacon à son goût;* let us differ at some of these points, but let us all work for the advancement of that art (it is not yet a science) which is so dear to our hearts.

